# Chloroplast genomic comparison provides insights into the evolution of seagrasses

**DOI:** 10.1186/s12870-023-04119-9

**Published:** 2023-02-22

**Authors:** Jun Chen, Yu Zang, Shuai Shang, Zhibo Yang, Shuo Liang, Song Xue, Ying Wang, Xuexi Tang

**Affiliations:** 1 College of Marine Life Sciences, Ocean University of China, Qingdao, Shandong China; 2 Ministry of Natural Resources, Key Laboratory of Marine Eco-Environmental Science and Technology, First Institute of Oceanography, Qingdao, Shandong China; 3 Laboratory for Marine Ecology and Environmental Science, Qingdao National Laboratory for Marine Science and Technology, Qingdao, Shandong China

**Keywords:** Seagrass, Chloroplast genome, Repeats, Phylogenetic analysis, Adaptive evolution

## Abstract

**Background:**

Seagrasses are a polyphyletic group of monocotyledonous angiosperms that have evolved to live entirely submerged in marine waters. Thus, these species are ideal for studying plant adaptation to marine environments. Herein, we sequenced the chloroplast (cp) genomes of two seagrass species (*Zostera muelleri* and *Halophila ovalis*) and performed a comparative analysis of them with 10 previously published seagrasses, resulting in various novel findings.

**Results:**

The cp genomes of the seagrasses ranged in size from 143,877 bp (*Zostera marina*) to 178,261 bp (*Thalassia hemprichii*), and also varied in size among different families in the following order: Hydrocharitaceae > Cymodoceaceae > Ruppiaceae > Zosteraceae. The length differences between families were mainly related to the expansion and contraction of the IR region. In addition, we screened out 2,751 simple sequence repeats and 1,757 long repeat sequence types in the cp genome sequences of the 12 seagrass species, ultimately finding seven hot spots in coding regions. Interestingly, we found nine genes with positive selection sites, including two ATP subunit genes (*atpA* and *atpF*), three ribosome subunit genes (*rps4*, *rps7*, and *rpl20*), one photosystem subunit gene (*psbH*), and the *ycf2*, *accD*, and *rbcL* genes. These gene regions may have played critical roles in the adaptation of seagrasses to diverse environments. In addition, phylogenetic analysis strongly supported the division of the 12 seagrass species into four previously recognized major clades. Finally, the divergence time of the seagrasses inferred from the cp genome sequences was generally consistent with previous studies.

**Conclusions:**

In this study, we compared chloroplast genomes from 12 seagrass species, covering the main phylogenetic clades. Our findings will provide valuable genetic data for research into the taxonomy, phylogeny, and species evolution of seagrasses.

**Supplementary Information:**

The online version contains supplementary material available at 10.1186/s12870-023-04119-9.

## Background

Along with coral reefs and mangroves, seagrass beds are one of the three main marine ecosystems, providing important habitats for marine life. They have significant ecological functions, such as stabilizing coastal sediments, providing nursery grounds for juvenile fish, and sequestering carbon [1–4]. The currently recognized seagrasses consist of approximately 74 species of six families and 13 genera in the order Alismatales, accounting for less than 1% of all flowering plant species [5–7]. Les et al. (1997) detected three separate origins of seagrasses using the *rbcL* gene, which was confirmed in subsequent studies [7–9]. Through independent evolutionary routes, seagrasses growing in intertidal and subtidal zones are characterized by similar environments, such as high salinity, low light, anaerobic soils, and extreme tides. Seagrasses have evolved shared traits such as salt tolerance, slender and soft leaves, carbon-concentrating mechanisms, and aerenchyma in the roots and rhizomes [10–14].

The unique evolutionary characteristics of seagrasses are similar to those of whales, which evolved from the land to aquatic environments [15]. The emergence of seagrasses is one of the most notable evolutionary transformations in the history of angiosperms. Recently, the broad application of technologies for genome sequencing has been demonstrated to be a valuable phylogenetic and evolutionary tool for revealing the genetic development and adaptive evolutionary mechanism of seagrasses to the marine environment [14, 16, 17]. For example, the first complete genome sequence and detailed genomic analysis of *Zostera marina* found that it had discarded several key innovative features of angiosperms, such as genes involved in stomatal and ethylene pathways, terpenoid synthesis, ultraviolet protection and far-infrared sensing, during its secondary entry into the sea, and also that it had recoded its cell wall components and expanded its sucrose synthesis and transport, ion transport and light-harvesting chlorophyll b-protein genes to adapt to the complex marine environment [17]. Meanwhile, Lee et al. (2016) found that genes related to hormone biosynthesis signal transduction and cell wall catabolism were also lost or modified in *Zostera muelleri* in the process of adapting to the marine environment [16]. Moreover, Lee et al. (2018) also sequenced the genomes of *Halophila ovalis* and explored the convergent evolutionary features with the above two seagrasses, finding that all three seagrasses lost genes related to ethylene and terpenoid biosynthesis while retaining those related to salt tolerance [14]. However, compared with higher terrestrial plants, genomics analysis in seagrasses is still in its infancy, and further research is urgently needed.

Plant chloroplasts are the main functional organs of plants, having been formed through endosymbiosis between early plants and cyanobacteria [18], and have multiple functions in plant cells, including playing a crucial role in photosynthesis and carbon fixation [19, 20]. Since the process of photosynthesis is influenced by three main factors (light irradiance and wavelength, carbon dioxide concentration, and temperature), it is reasonable to assume that the genetic basis of the cp genome changes in response to the conversion of living habitats. Chloroplasts are essential organelles in plant evolution due to their features. The cp genome is small and contains well-characterized features, including a relatively stable gene content and conserved structural features such as a large single copy (LSC) region, a small single copy (SSC) region, and a pair of inverted repeats (IRs) regions, together with the feature of a slow rate of nucleotide substitution, providing essential information to support comparative evolutionary research [19, 21, 22]. Adaptive evolution is defined as the improved adaptability of a species to changing environmental conditions. Given the conservation of the cp genome, observable alterations should be anticipated if adaptive evolution occurs in this molecule. Thus, a comparative analysis of the cp genomes of seagrasses should enhance our understanding of plant adaptation to the sea environment.

In the present study, we aimed to provide comprehensive insights into the evolution of cp genomes across prominent seagrass families. Here, we assembled and characterized the cp genome sequences of two seagrass species and compared them with 10 published seagrass cp genomes from four families, including Cymodoceaceae, Ruppiaceae, Zosteraceae, and Hydrocharitaceae. We also identified repeat sequences and positive selection, and reconstructed the phylogenetic relationships and molecular divergence dates for the major lineages within the seagrasses. These results will help develop genetic markers for seagrasses and provide an important theoretical basis for subsequent population genetic analysis and phylogenetic relationship analysis.

## Results

### Genomic features of seagrasses

**Fig. 1:**
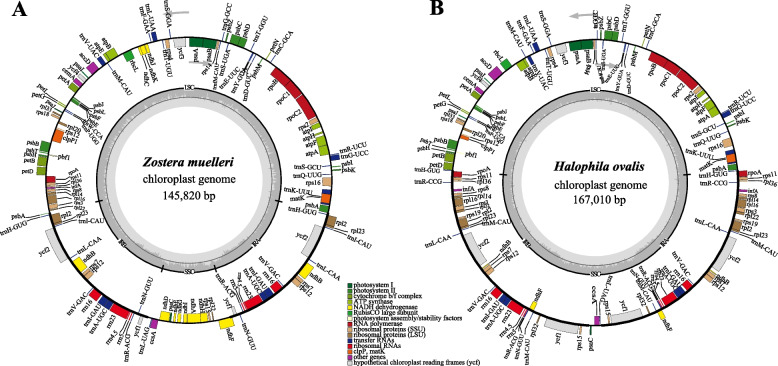
Chloroplast genome gene map of *Halophila ovalis* and *Zostera muelleri*. Genes on the inside of the outer circle are transcribed clockwise, while those outsides are transcribed counterclockwise

This study analyzed 12 cp genomes covering the four families represented by seagrasses: Zosteraceae, Hydrocharitaceae, Ruppiaceae, and Cymodoceaceae. Among them, *Z. muelleri* and *H. ovalis* were assembled and annotated for the first time in this study (Fig. 1). All cp genomes had a typical four-part structure: a LSC, an SSC, and two IRs (Table 1). The length of the 12 cp genomes ranged from 143,877 bp to 178,261 bp and varied in size among the different families as follows: Hydrocharitaceae > Cymodoceaceae > Ruppiaceae > Zosteraceae. The IR was 24,399–44,815 bp in length, the LSC was 78,949–89,851 bp in length, and the SSC was 2,150–19,160 bp in length. The length differences between families were mainly related to the expansion and contraction of the IR region. The cp genomes of the seagrass species had 116–158 genes, including 78–122 protein-coding genes, eight ribosomal RNA genes (rRNA), and 30–42 transfer RNA genes (tRNA). The total GC content of the cp genomes was 35.5%–39.2%, and the GC content was more similar for species of the same family.

**Table 1:** Chloroplast genome features of 12 seagrass species

Family	Species Name	Genome size (bp)	Reference	LSC size (bp)	IR size (bp)	SSC size (bp)	GC (%)	Total N.o of genes	PCGs N.o	tRNA N.o	rRNA N.o
Zosteraceae	*Zostera nigricaulis*	145,883	NC_058621.1	83,541	24,430	13,482	35.9	131	85	38	8
	*Phyllospadix iwatensis*	152,726	NC_058622.1	84,753	25,167	17,639	36.2	132	86	38	8
	*Zostera japonica*	146,090	NC_058623.1	83,664	24,628	13,169	35.9	127	85	34	8
	*Zostera marina*	143,877	NC_036014.1	83,224	25,915	8,823	35.5	116	78	30	8
	*Zostera muelleri*	145,820	In this study	83,524	24,399	13,498	35.9	131	85	38	8
Hydrocharitaceae	*Enhalus acoroides*	176,211	MN736636.1	89,851	42,105	2,150	38.4	142	95	39	8
	*Thalassia hemprichii*	178,261	NC_043774.1	83,691	44,815	4,940	39.2	158	122	28	8
	*Halophila beccarii*	168,585	NC_051970.1	80,881	41,487	4,730	38.5	132	88	36	8
	*Halophila ovalis*	167,010	In this study	78,949	42,898	2,265	38.8	140	90	42	8
Ruppiaceae	*Ruppia brevipedunculata*	158,943	MN736637.1	88,857	25,478	19,130	35.8	132	87	37	8
	*Ruppia sinensis*	158,897	MN233650.1	88,952	25,449	19,047	35.9	136	88	40	8
Cymodoceaceae	*Syringodium isoetifolium*	159,333	MZ325253.1	89,055	25,559	19,160	35.9	131	86	37	8

### Repeat sequence analysis

**Fig. 2:**
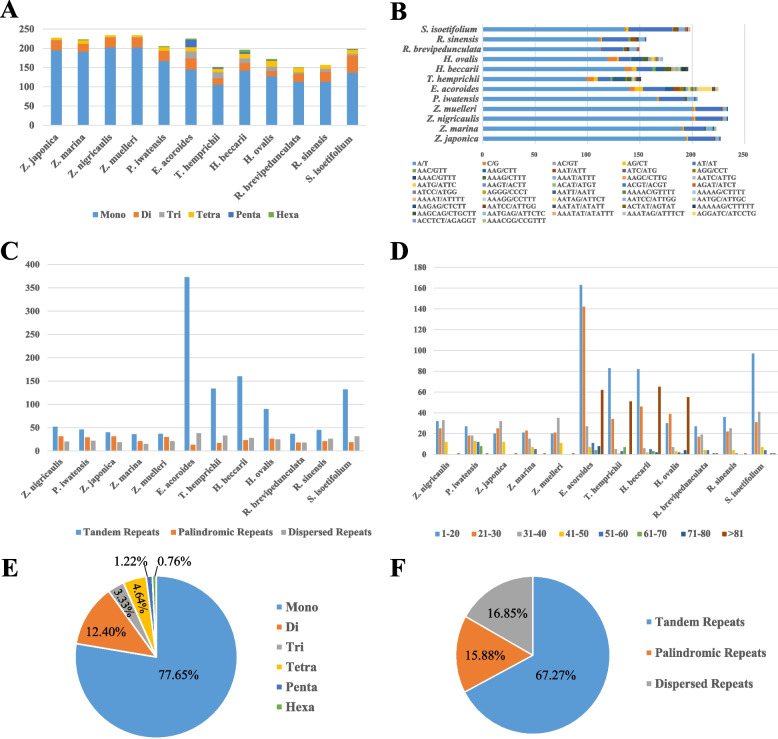
Analysis of the repeated sequences of the 12 chloroplast genomes of seagrasses compared in this study. **A**: The number of SSRs in six SSR types; **B**: summary of different SSR repeat unit types; **C**: the number of repeat elements in three long repeat types; **D**: summary of long repeat types by length; **E**: distribution of SSR types; and **F**: distribution of long repeat types

In this study, a total of 2,751 simple sequence repeats (SSRs) were identified in the cp genome sequences of the 12 seagrass species (Fig. 2). Among them, the highest percentages of SSRs were mononucleotides (77.65%), followed by dinucleotides (12.40%), tetranucleotides (4.64%), trinucleotides (3.33%), pentanucleotides (1.22%), and hexanucleotides (0.76%) (Fig. 2E). Furthermore, we found that the Zosteraceae species had more SSRs, and A/T was the main mononucleotide SSR type in all species (Fig. 2B). In addition, we identified 1,757 long repeat sequence types in the cp genome sequences of the 12 seagrass species, including 1,182 tandem repeats, 279 palindromic repeats, and 296 dispersed repeats, respectively (Fig. 2C). The lengths of these long repeats were highly variable, with repeats of 1–20, 21–30, and 31–40 bp being more abundant among the three repeat types (Fig. 2D). Among them, Hydrocharitaceae species had more long repeat sequences (especially tandem repeats) and > 81 bp repeat types.

### Comparative analysis

**Fig. 3:**
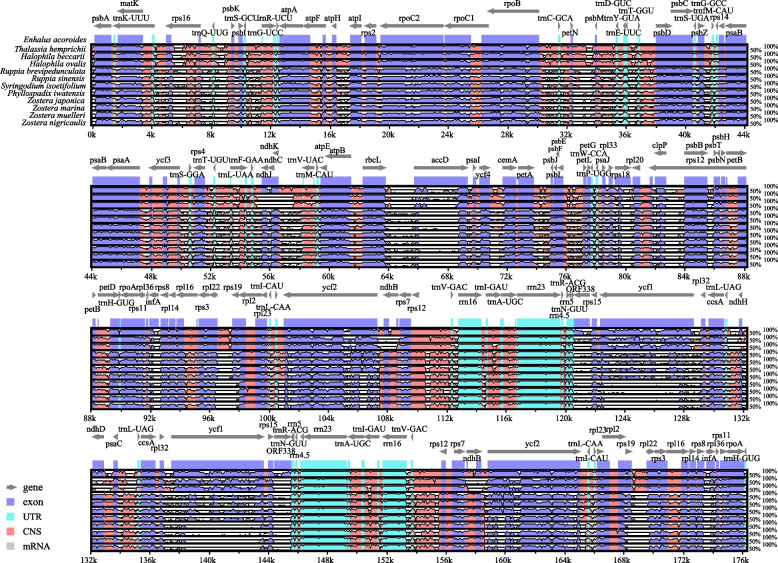
Visualization of the alignment of 12 chloroplast genome sequences of seagrasses. The chloroplast genome of *Enhalus acoroides* was used as the reference. The* Y*-axis depicts percent identity to the reference genome (50–100%) and the* X*-axis depicts sequence coordinates within the chloroplast genome

The overall sequence identity of the cp genomes of 12 seagrass species was visualized using the mVISTA program, using the *E. acoroides* annotated sequences as a reference. Figure 3 shows a genome-wide alignment with high sequence similarity (> 90% identity). The cp genomes of the same family showed higher similarity. The divergence level of the non-coding regions was higher than the coding regions. Additionally, the LSC and SSC regions showed a higher level of sequence divergence than the IR regions. In addition, 59 coding regions were extracted to calculate the nucleotide variability (Supplementary Fig. 1), and the loci with the largest variation were *accD*, *clpP*, *infA*, *rpl22*, *rps15*, *rps18*, and *ycf1*.

### Phylogenetic relationships

**Fig. 4:**
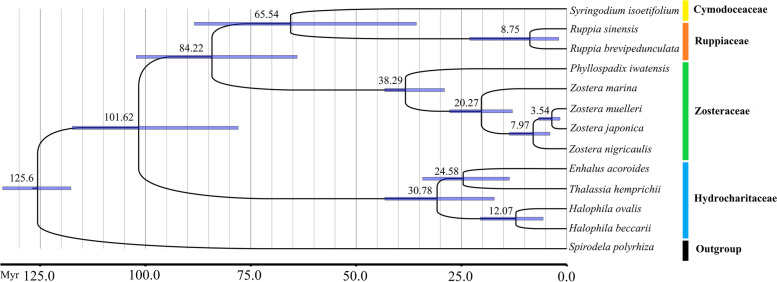
Phylogenetic ML tree and time-calibrated phylogenetic tree of 59 single copy genes of 12 seagrasses

To investigate the phylogenetic relationships of the seagrasses, we constructed a maximum likelihood (ML) tree using RAxML (Fig. 4). As indicated in the tree, the shared nucleotide-coding genes were divided into four major clades: Zosteraceae, Cymodoceaceae, Hydrocharitaceae, and Ruppiaceae, among which Cymodoceaceae and Ruppiaceae had a relatively close relationship. Moreover, *Z. muelleri* was more closely related to *Z. japonica*, followed by *Z. nigricaulis* and *Z. marina* within Zosteraceae. These results showed that phylogenetic proximity was associated with the traditional taxonomic group. The results of the MCMCtree analysis of species divergence times in seagrasses are also shown in Fig. 4. The results showed that the divergence time between *S. polyrhiza* and seagrasses was about 125.6 Mya (95% PHD = 117.7–133.9 Mya, calibration point = 128.0 Mya). The diversification of Hydrocharitaceae, Zosteraceae, Ruppiaceae, and Cymodoceaceae in Alismatales was about 65.54–101.62 Mya. Within Zosteraceae, the divergence between *Phyllospadix* and *Zostera* was approximately 38.29 Mya. The divergence time of *Z. marina* (mean age of 20.27 Mya) was much earlier than that of *Z. nigricaulis* (mean age = 7.97 Mya), and that of *Z. muelleri* and *Z. japonica* (mean age = 3.54 Mya).

### Adaptive evolution analysis

Using the M8 model and the Datamonkey web server SLAC, FEL, and MEME methods, a total of nine genes were detected with positive selection sites (Supplementary Table S1), including two ATP subunit genes (*atpA* and *atpF*), two ribosome small subunit genes (*rps4* and *rps7*), one photosystem subunit gene (*psbH*), one ribosome large subunit gene (*rpl20*), and the *ycf2*, *accD*, and *rbcL* genes. Among them, the *ycf2* gene harbored eight sites under positive selection, with two in *accD*, while the other seven genes each had only one positively selected site.

## Discussion

### Chloroplast sequence variation

The cp genomes of the 12 seagrasses in this study ranged in size from 143,877 to 178,261 bp, which is similar to most angiosperms [23]. However, the size of the cp genome within the seagrasses varied widely, with those of the species of Hydrocharitaceae being much larger than those of the other three families (Table 1). These differences in cp genome size may have been due to the expansion and contraction of the border positions between the IR and SSC regions [24, 25]. The GC content acts as a landmark for the physical location of functional elements in the genome [26] and is positively correlated with the rate of recombination and exon density [27]. Generally, cp genomes are characterized by a low GC content; however, in this study, Hydrocharitaceae had a higher GC content (38.4%–39.2%) than other seagrasses. Moreover, we used mVISTA to compare the whole cp of 12 species of seagrasses and used DnaSP to analyze the percentage of variable loci in 59 single-copy shared genes. Similar to previous results, the variation in noncoding regions was more significant than that of the coding regions [24, 28]. Genes with a relatively high mutation rate can be used as DNA barcodes to help distinguish between accessions within a given taxon and varieties in germplasm resources [29, 30]. We detected seven hot spots in coding regions, which can be used as candidate DNA barcodes for assessing the phylogenetic relationships and interspecific differences among seagrasses.

In addition, the 12 cp genomes of the seagrasses contained a high frequency of large repeats. Previous studies have suggested that larger and complex repeat sequences have played key roles in sequence rearrangements and cp genome evolution [31, 32]. In this study, we found that the Hydrocharitaceae genomes had the highest numbers of repeats and a significant correlation (*R* = 0.73, *P* = 0.0065) between the size of the cp genomes and the number of repeats (Supplementary Fig. 2). Furthermore, tandem repeats were the most abundant type of repeat in the Hydrocharitaceae cp genomes, which differs from the high content of dispersed repeats reported in other angiosperm lineages [33, 34]. From an evolutionary perspective, variations in repeated sequences among species are due to natural selection and adaptation by organisms to diverse environments [35]. Previous studies have shown that cp SSRs are dominated by A/T repeats, which contribute significantly to the AT richness of cp genomes [36]. In this study, SSRs in the seagrass cp genome also contained many AT units, and mononucleotide repeats accounted for 77.65% of the total SSRs. In addition, we found that dinucleotide repeats (AT/AT) were the most common of the different types in all the seagrasses, while pentanucleotide and hexanucleotide SSRs were rarely found. The copy number variation of SSRs in the cp genome was highly polymorphic, and these seagrass variants can be used as molecular genetic markers for future studies in population genetics, phylogeography, phylogenetics, and species identification [37–39].

### Adaptive selection

In our study, we identified nine genes with positive selection sites. Among them, ATP synthase is essential for plant photosynthesis and is usually a product of two genetic systems in plants [40]. In this study, we found that two ATP subunit genes (*atpA* and *atpF*) exhibited site-specific selection. Additionally, three genes (*rps4*, *rps7*, and *rpl20*) encoding ribosomal subunits were identified under positive selection. One photosystem II subunit gene (*psbH*) was also under positive selection. ACCase is a key enzyme in fatty acid biosynthesis [41]. The β-CT subunit of ACCase is encoded by *accD*, which is the only component of plant lipid metabolism known to be encoded by the plastid genome [42, 43]. We identified positively selected sites (PSSs) in *accD*, which may have played key roles in seagrass fatty acid biosynthesis. Additionally, *rbcL* provides all the catalytically essential residues of RuBisCO, a critical enzyme for both the reductive and oxidative photosynthetic carbon cycles. A previous study showed that many aquatic plants had acquired carbon-concentrating mechanisms to overcome the potentially low, fluctuating supply of CO_2_ for underwater photosynthesis [44]. In particular, the *rbcL* gene evolved under positive selection in *Potamogeton* [45]*.* Although seagrasses can also use both CO_2_ and HCO_3_^−^ (bicarbonate) for photosynthetic carbon reduction [46], they live mainly in seawater with low CO_2_ concentration, and so positive selection of this gene may be involved in increasing their CO_2_ utilization efficiency. We also found that *ycf2* had eight sites under positive selection. This gene is the largest cp gene reported in angiosperms and is valuable for assessing sequence variations and evolutionary processes in plants [24, 47, 48]. Positive selection on the *ycf2* gene has also been found in the adaptive evolution of other species [28, 49, 50]. In short, these positively selected genes may play a key role in the adaptation of seagrasses to the marine environment.

### Phylogenetic relationships

Seagrasses are a paraphyletic group of marine angiosperms that evolved in parallel three to four times from land plants back to the sea [7, 17]. Therefore, seagrass is a generic term for a variety of aquatic angiosperms and therefore represents an ecological group rather than a taxonomic group. In this study, the phylogenetic trees of the seagrasses based on the whole-genome nucleotide coding sequences of the cp genomes were clearly divided into four major clades, representing Zosteraceae, Cymodoceaceae, Hydrocharitaceae, and Ruppiaceae, which is similar to previous analyses based on various molecular datasets [7, 24, 51–53]. Divergence time estimation dated the divergence of seagrasses and *S. polyrhiza* at around 125.6 Mya. This age is consistent with previous genome-based studies [17, 54]. The stem node ages of the seagrass lineages Zosteraceae and Cymodoceaceae/Ruppiaceae were at 84.22 Mya, which is slightly older than that in recent studies [52, 55]. The divergence time of *E. acoroides*/*T. hemprichii* and *Halophila* within Hydrocharitaceae was around 30.78 Mya. This age is slightly older than that reported by Li et al. (2012) at around 19.41 Mya [56], but is younger than that reported by Li et al. (2022) at around 34.64 Ma [52]. Among seagrasses, all species of Zosteraceae are seagrasses, whereas other families contain more than just seagrass species. Among Zosteraceae, the genus *Phyllospadix* has a clear taxonomic status owing to its morphological features and chromosome number that differs significantly from other species [57–59]. In this study, we also found that *P. iwatensis* was the first to diverge from the species of the Zosteraceae family in 38.29 Ma. This age is consistent with previous phylogenetic analyses and molecular clock estimates based on the cp *rbcL* and *matK* loci, which suggested that the family Zosteraceae emerged about 100 Ma, and the divergence of *Zostera* and *Phyllospadix* began around 36 Ma [60]. Fossil evidence indicates that seagrasses originated in the Late Cretaceous [61]. As higher angiosperms, seagrasses have an existing root system and can occupy a previously empty niche in shallow sedimentary shoreline marine systems. This may have been facilitated by the Cretaceous-Paleogene extinction event, which took place roughly 70–65 Mya and coincided with a lineage-specific whole-genome duplication in *Zostera* [17].

## Conclusions

In this study, we sequenced the cp genomes of two seagrass species (*Z. muelleri* and *H. ovalis*) and revealed the cp genomic features with the available seagrass genomes obtained from the NCBI database. We screened out 2,751 SSRs and 1,757 long repeat sequence types in the cp genome sequences of the 12 seagrass species. We also identified nine positive selection genes and seven variable regions, which provide a reference for developing DNA markers and evaluating adaptive evolution in further studies of seagrass species. Phylogenetic and divergence time analysis based on the current data was generally consistent with previous studies. These findings will be valuable for further study of the cp genomes of seagrass species, and will provide valuable resources for studies of plant adaptation to marine environments.

## Methods

### Sampling, DNA extraction, and sequencing

In this study, two seagrasses, *Z. muelleri* and *H. ovalis*, were collected from their natural habitats, and the collection processes of both conformed to local and national regulations. The voucher specimens of *Z. muelleri* (voucher number: OUC-S120) and *H. ovalis* (voucher number: OUC-S121) were deposited in the Herbarium of Marine Ecology Laboratory, College of Marine Life Sciences, Ocean University of China (OUC). The samples were identified by Tang Xuexi, a Professor at OUC. Fresh leaves (100 mg) were preserved in silica gel immediately after sampling, after which TRIzol® reagent (Invitrogen, USA) was used in the laboratory to extract their total DNA following the manufacturer’s protocol. The integrity, quality, and concentration of the DNA were determined by 1% agarose gel electrophoresis and a NanoDrop Spectrophotometer 2000 (Thermo Fisher Scientific, Waltham, MA, USA). Illumina TruSeq™ Nano DNA Sample Prep Kits were used for Illumina sequencing library construction, and the DNA was sequenced using an Illumina HiSeq 4000 platform (150 bp*2). After Illumina sequencing, approximately 63,351.8 Mb and 97,155.8 Mb of raw data for *Z. muelleri* and *H. ovalis* were generated, and these raw reads were QC-filtered and trimmed using the Trimmomatic 0.39 software [62]. In total, 61,803.7 Mb and 91,116.5 Mb clean data were obtained. Afterwards, *Z. marina* was used as a reference sequence to assemble its genome using the NOVOPlasty v2.7.2 software [63], and the GapCloser software [64] was used to fill in the remaining local internal gaps and correct for single-base polymorphisms. Finally, the starting position and orientation of the chloroplast assembly sequence were determined using the reference genome, and the possible partition structure of the chloroplast (LSC/IR/SSC) was determined to obtain the final chloroplast genome sequence.

### Genome annotation and comparative analysis

The assembled cp genomes were annotated using GeSeq software [65], with the following parameters: (1) protein search identity, 60; (2) rRNA, tRNA, DNA search identity, 35; and (3) third-party tRNA annotators, the tRNAscan-SE software [66]. Then, the annotation results were checked with BLAST and DOGMA [67]. The circular maps of the cp genomes of the two seagrass samples were presented using Organellar Genome DRAW software [68]. Subsequently, two complete cp genomes were deposited in GenBank with the following accession numbers: *Z. muelleri* (OP611572) and *H. ovalis* (OP611573). Alignments of the 12 complete cp seagrass genome sequences (Table 1) were visualized using mVISTA [69]. Finally, all shared coding regions of the extracted 12 seagrass cp genomes were aligned separately using MUSCLE [70], and the nucleotide variability of each selected region was evaluated separately using DNASP v5.10 [71].

### Repeat sequence analysis

Repeat sequences within the seagrass cp genome, including forward (F), reverse (R), palindrome (P), and complement (C), were searched using the online software REPuter (https://bibiserv.cebitec.uni-bielefeld.de/reputer) [72], and identified with the following conditions: minimal size of 30 bp; 90% or greater sequence identity; and Hamming distance equal to 3. Tandem Repeats Finder v4.09 software (https://tandem.bu.edu/trf/trf.html) [73] was used to detect tandem repeats with > 6 bp repeat units. SSR markers were identified in the 12 seagrasses sequences using MISA (http://pgrc.ipk-gatersleben.de/misa/misa.html) [74] with a motif size of 1–6 nucleotides and thresholds of eight, five, four, three, three, and three repeat units for mono-, di-, tri-, tetra-, penta-, and hexanucleotide SSRs, respectively.

### Phylogenetic relationships and divergence time estimation

Phylogenetic analysis of the 12 cp genomes of the seagrass species was performed with *Spirodela polyrhiza* as the outgroup. All shared nucleotide-coding sequences were concatenated into a super matrix and aligned with MUSCLE [70]. A phylogenetic tree was produced using the ML method based on the GTRGAMMA model with 1,000 bootstrap replicates using RAxML [75]. MCMCtree implemented in the PAML 4.7 package was used to estimate the speciation time [76]. The obtained ML tree was used as the input tree file for the analysis process. Three calibration points (*E. acoroides* vs. *T. hemprichii*: 8.59–34.64 MYA, *Zostera* vs. *P. iwatensis*: 15.29–42.00 MYA, and Zosteraceae vs. P. Hydrocharitaceae: 72.70–117.00 MYA) derived from the TimeTree database (http://www.timetree.org/) were applied to constrain the divergence times of the nodes.

### Adaptive evolution analysis

The CODEML program in the PAML 4.7 package [76] was used to calculate the rate of nonsynonymous substitutions (*dN*) and synonymous substitutions (*dS*) of single-copy protein coding genes (PCGs). The *dN*/*dS* ratio, also known as the *ω* value, was used to measure the rate of gene evolution, where *ω* values greater than 1, equal to 1, and less than 1 represent positive selection, neutral selection and purifying selection, respectively. The site model allows for different ω values for different sites in the same sequence. The M7 (null hypothesis: 0 < *ω* < 1) and M8 (alternative hypothesis: *ω* > 1) models were used to detect PSSs on 12 seagrass species. In the likelihood ratio test (LRT) results of the two models, the alternative hypothesis M8 model was accepted if *p* < 0.05, and the null hypothesis M7 model was accepted if this was not true. With *p* < 0.05, the PSSs detected using the M8 model were considered as potential PSSs when the Bayes empirical Bayes (BEB) posterior probability was > 0.90 [77]. Meanwhile, PSSs were identified using the Data Monkey Web Server (http://www.datamonkey.org/) [78] based on three methods, namely the FEL, SLAC, and MEME models, with *P* < 0.1. In this study, positive selection sites detected using at least three or more of the methods above were considered to be positive selection sites.

## Supplementary Information

**Additional file 1: Supplementary Figure 1.** Percentages of variable characters in protein-coding regions among the 12 chloroplast genomes of the seagrasses.

**Additional file 2: Supplementary Figure 2.** Relationships between seagrass chloroplast genome sizes and the number of repeats.

**Additional file 3: Table S1.** Positively selected sites of 59 single-copy genes shared by twelve seagrass species.

## Data Availability

All newly sequenced chloroplast genomic sequences of the two seagrasses in this study can be downloaded from the National Center for Biotechnology Information (NCBI) under accession numbers OP611572 (https://www.ncbi.nlm.nih.gov/nuccore/OP611572) and OP611573 (https://www.ncbi.nlm.nih.gov/nuccore/OP611573). Information on the chloroplast genomic sequences of the other 10 seagrasses used for the combined analysis can also be downloaded from GenBank and found in Table 1.

## Abbreviations

cp: Chloroplast
LSC: Large single copy
SSC: Small single copy
IRs: Inverted repeats
SSR: Simple sequence repeat
ML: Maximum likelihood
*dN*: Nonsynonymous substitutions
*dS*: Synonymous substitutions
LRT: Likelihood ratio test
BEB: Bayes empirical Bayes
rRNA: Ribosomal RNA genes
tRNA: Transfer RNA genes
PCGs: Protein coding genes
PSSs: Positively selected sites
