# Supplementary material for: Chloroplast genomic comparison provides insights into the evolution of seagrasses
Source: BMC Plant Biol. 2023 Feb 22;23:104. doi: 10.1186/s12870-023-04119-9 (PMC9945681; doi:10.1186/s12870-023-04119-9)
Supplement: Supplementary file 1 — Additional file 1: Supplementary Figure 1. Percentages of variable characters in protein-coding regions among the 12 chloroplast genomes of the seagrasses. [file 12870_2023_4119_MOESM1_ESM.docx]

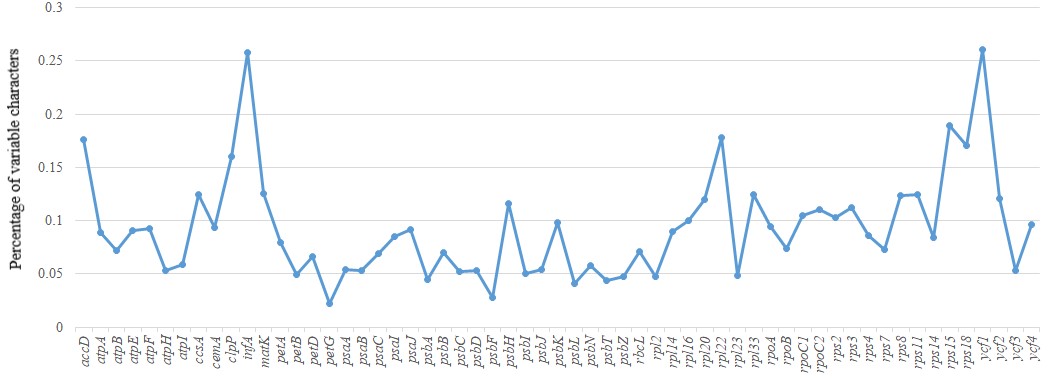


**Supplementary Figure 1.** Percentages of variable characters in protein-coding regions among the 12 chloroplast genomes of the seagrasses.
