# Supplementary material for: Chloroplast genomic comparison provides insights into the evolution of seagrasses
Source: BMC Plant Biol. 2023 Feb 22;23:104. doi: 10.1186/s12870-023-04119-9 (PMC9945681; doi:10.1186/s12870-023-04119-9)
Supplement: Supplementary file 2 — Additional file 2: Supplementary Figure 2. Relationships between seagrass chloroplast genome sizes and the number of repeats. [file 12870_2023_4119_MOESM2_ESM.docx]

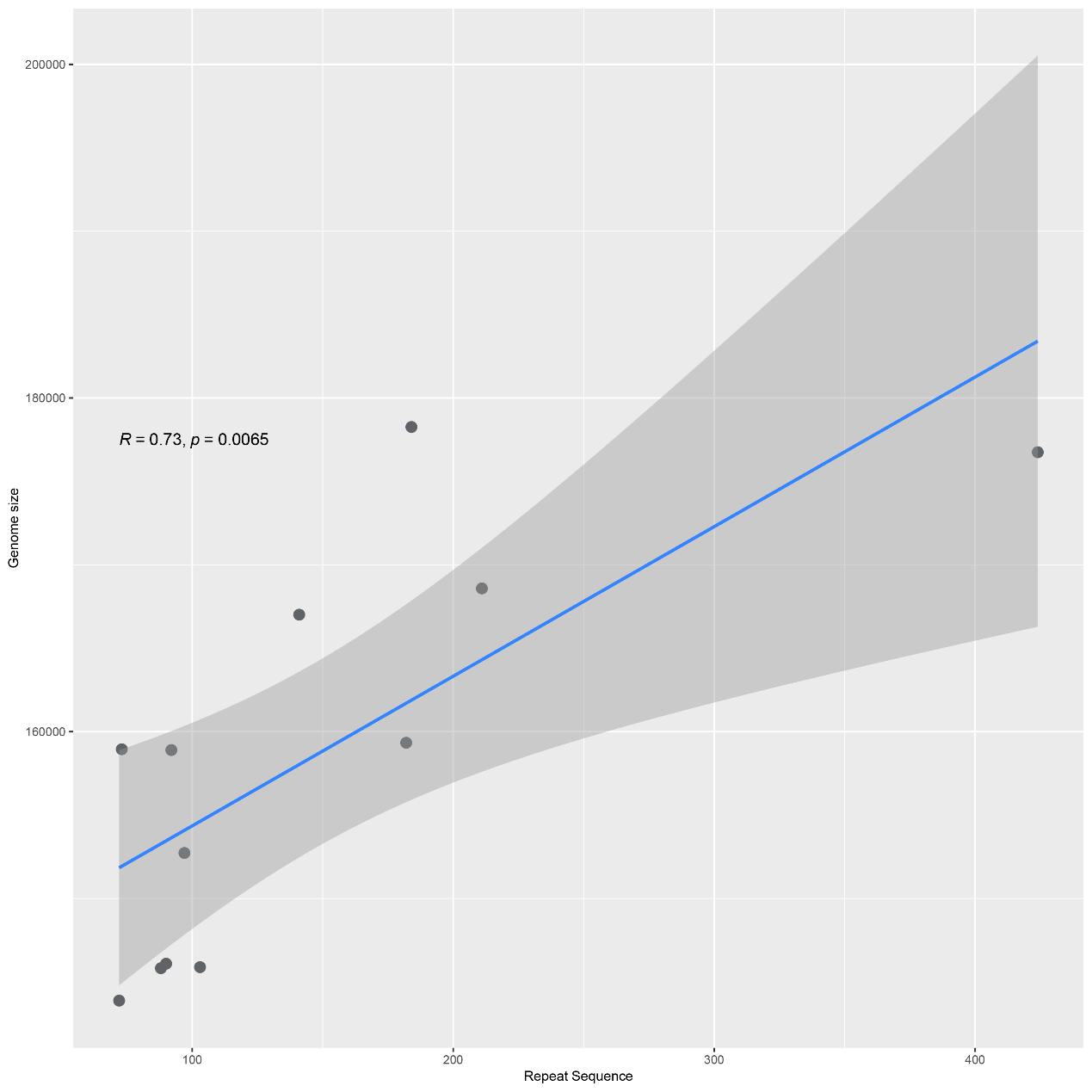


**Supplementary Figure 2.** Relationships between seagrass chloroplast genome sizes and the number of repeats.
