# Supplementary material for: Chloroplast genomic comparison provides insights into the evolution of seagrasses
Source: BMC Plant Biol. 2023 Feb 22;23:104. doi: 10.1186/s12870-023-04119-9 (PMC9945681; doi:10.1186/s12870-023-04119-9)
Supplement: Supplementary file 3 — Additional file 3: Table S1. Positively selected sites of 59 single-copy genes shared by twelve seagrass species. [file 12870_2023_4119_MOESM3_ESM.docx]

**Table S1** Positively selected sites of 59 single-copy genes shared by twelve seagrass species.

| Gene | *Ln L* M7 | *Ln L* M8 | *P-value* | M8 | SLAC | FEL | MEME |
| --- | --- | --- | --- | --- | --- | --- | --- |
| *psbA* | -2209.51 | -2207.57 | 0.143 | NA | NA | NA | NA |
| *matK* | -5351.32 | -5348.51 | 0.0602 | NA | NA | 46 | 62 66 137 155 362 465 479 |
| *psbK* | -460.89 | -460.81 | 0.923 | NA | NA | NA | NA |
| *psbI* | -228.62 | -228.62 | 1 | NA | NA | NA | NA |
| *psbD* | -2280.22 | -2278.12 | 0.122 | NA | NA | 4 9 | 4 9 11 |
| *psbC* | -2947.65 | -2947.65 | 1 | NA | NA | NA | 349 |
| *rpoB* | -8971.14 | -8964.94 | 0.00203** | NA | NA | 567 746 801 | 210 242 404 567 583 588 718 746 761 801 |
| *rpoC1* | -6163.14 | -6149.43 | 1.11E-06** | 142 162 163 296 | NA | 14 306 | 14 49 110 142 162 163 201 226 306 363 559 571 572 589 599 |
| *rpoC2* | -12632.25 | -12629.35 | 0.0550 | NA | NA | 450 664 829 898 | 216 659 744 811 829 898 966 972 1217 1261 1314 1319 |
| *rps2* | -2024.83 | -2024.55 | 0.756 | NA | NA | 113 | 113 146 159 |
| *atpI* | -1661.32 | -1660.99 | 0.719 | NA | NA | NA | 101 |
| *atpH* | -506.32 | -506.32 | 1 | NA | NA | NA | NA |
| *atpA* | -4318.61 | -4314.67 | 0.0194* | 61 253 | ***383*** | ***383*** | 5 33 ***383*** 406 459 |
| *atpF* | -1625.71 | -1615.51 | 3.71E-05** | ***49*** 50 | ***49*** | ***49*** | 31 ***49*** 50 51 53 59 92 110 114 |
| *psbZ* | -352.37 | -352.37 | 1 | NA | NA | NA | NA |
| *rps14* | -834.84 | -834.21 | 0.532 | 11 | NA | 65 | 65 |
| *psaB* | -4826.45 | -4826.27 | 0.835 | NA | NA | 150 | 282 600 612 731 |
| *psaA* | -5043.21 | -5043.14 | 0.932 | 25 | NA | NA | 37 620 |
| *ycf3* | -1075.02 | -1075.02 | 1 | NA | NA | NA | NA |
| *rps4* | -1690.55 | -1685.87 | 0.00927* | 43 ***158*** | NA | 42 ***158*** | 42 43 148 ***158*** |
| *atpE* | -1100.34 | -1099.78 | 0.571 | NA | NA | NA | 79 |
| *atpB* | -3582.48 | -3582.31 | 0.843 | NA | NA | NA | 114 201 359 |
| *rbcL* | -3572.32 | -3569.79 | 0.0796 | ***449*** | NA | ***449*** | 93 131 224 355 375 ***449*** 456 |
| *accD* | -3459.13 | -3447.52 | 9.07E-06** | 14 68 ***76*** ***220*** | ***76*** | ***76*** ***220*** 255 | 15 18 29 71 ***76*** 77 93 219 ***220*** 224 255 257 |
| *psaI* | -286.64 | -286.54 | 0.905 | NA | NA | NA | 29 |
| *ycf4* | -1533.49 | -1533.49 | 1 | NA | NA | NA | 26 135 |
| *cemA* | -1728.91 | -1728.91 | 1 | NA | NA | 24 | NA |
| *petA* | -2515.96 | -2515.96 | 1 | NA | NA | NA | 148 229 |
| *psbJ* | -277.93 | -276.97 | 0.383 | 25 | NA | NA | NA |
| *psbL* | -217.85 | -217.46 | 0.677 | NA | NA | NA | NA |
| *psbF* | -215.07 | -215.07 | 1 | NA | NA | NA | NA |
| *petG* | -188.37 | -188.37 | 1 | NA | NA | NA | NA |
| *psaJ* | -304.70 | -303.51 | 0.304 | NA | NA | NA | NA |
| *rpl33* | -676.63 | -676.63 | 1 | NA | NA | NA | 2 |
| *rps18* | -1278.63 | -1271.45 | 0.000762** | 16 18 91 92 93 94 97 | NA | 22 | 9 15 16 18 19 22 24 37 54 97 |
| *rpl20* | -1199.94 | -1192.73 | 0.00074** | ***75*** | NA | 23 ***75*** | 21 48 ***75*** 76 116 |
| *clpP* | -2591.96 | -2585.30 | 0.00128** | NA | NA | 127 128 184 | 81 127 128 129 131 132 183 184 185 |
| *psbB* | -3765.21 | -3764.06 | 0.317 | 352 | NA | NA | 31 132 |
| *psbT* | -191.13 | -191.13 | 1 | NA | NA | NA | NA |
| *psbH* | -643.11 | -640.98 | 0.119 | ***12*** | NA | ***12*** | ***12*** 15 |
| *petB* | -1413.28 | -1411.83 | 0.234 | NA | NA | 2 | 2 |
| *petD* | -1173.91 | -1173.52 | 0.677 | NA | NA | NA | 2 |
| *rpoA* | -2781.35 | -2781.35 | 1 | NA | NA | NA | 3 188 220 236 241 |
| *rps11* | -1228.15 | -1228.15 | 1 | NA | NA | NA | 51 107 |
| *infA* | -971.13 | -971.08 | 0.951 | NA | NA | NA | 29 73 |
| *rps8* | -1145.88 | -1144.78 | 0.333 | NA | NA | NA | 2 |
| *rpl14* | -971.81 | -971.81 | 1 | NA | NA | NA | NA |
| *rpl16* | -1211.90 | -1205.27 | 0.00132** | 1 133 | NA | 74 | 76 83 129 |
| *rps3* | -1975.42 | -1975.32 | 0.905 | NA | NA | 82 84 | 53 55 80 81 107 131 |
| *rpl22* | -565.82 | -555.31 | 2.72E-05** | 44 | NA | NA | 44 |
| *rpl2* | -1585.38 | -1582.94 | 0.0872 | 180 222 | NA | NA | 250 |
| *rpl23* | -614.55 | -611.64 | 0.0544 | 93 | NA | NA | 17 |
| *ycf2* | -15889.02 | -15754.27 | 0** | 4 50 103 ***139*** ***203*** 204 233 248 249 252 254 256 258 271 ***272*** 275 289 298 316 320 327 329 332 333 338 339 343 ***363*** 584 ***656*** ***663*** 669 684 685 686 692 812 925 ***956*** 964 965 ***983*** 1238 1373 1389 1397 1400 1401 1402 | NA | ***139*** ***203*** 246 269 ***272*** 310 326 332 343 ***363*** 622 642 ***656*** ***663*** 685 706 ***956*** ***983*** 1154 1285 1293 | 5 21 33 41 84 85 87 103 ***139*** 161 ***203*** 204 205 206 207 209 241 246 248 251 256 258 262 264 269 ***272*** 278 284 287 289 297 300 303 307 316 318 324 326 327 335 338 ***363*** 441 524 556 583 586 622 642 655 ***656*** 660 661 ***663*** 684 685 686 689 691 692 699 706 715 729 772 773 801 804 823 829 925 ***956*** 964 966 ***983*** 1057 1217 1218 1221 1222 1226 1229 1230 1231 1232 1234 1276 1284 1286 1293 1316 1317 1320 1356 1358 1373 1376 1389 1399 1401 |
| *rps7* | -1148.01 | -1144.22 | 0.0226* | 24 ***43*** | NA | 24 ***43*** | ***43*** 147 |
| *ccsA* | -3295.70 | -3292.21 | 0.0305* | 171 | NA | 223 | 35 117 223 259 286 303 |
| *psaC* | -599.31 | -599.07 | 0.787 | 70 | NA | NA | 6 70 |
| *rps15* | -806.88 | -806.88 | 1 | NA | NA | NA | 32 62 63 |
| *ycf1* | -1300.51 | -1296.65 | 0.0211* | NA | NA | NA | 5 6 27 38 |
| *psbN* | -292.05 | -291.96 | 0.914 | NA | NA | NA | NA |

Note: *Signiﬁcant at 5% level; ** signiﬁcant at 1% level; Underlined, italicized, and bold represent sites identified by at least three methods
